# Retraction Note: Enhancing blockchain transaction classification with ensemble learning approaches

**DOI:** 10.1038/s41598-026-44421-8

**Published:** 2026-03-16

**Authors:** Amrutanshu Panigrahi, Abhilash Pati, Bibhuprasad Sahu, Rourab Paul, Ajit Kumar Nayak, Subrata Chowdhury, Ramya Govindaraj, J Shreyas

**Affiliations:** 1 Department of CSE, Siksha ‘O’ Anusandhan (Deemed to be University), Bhubaneswar, Odisha India; 2 Department of Computer Science and Engineering, Shiv Nadar University, Chennai, Tamil Nadu India; 3 Department of Information Technology, Vardhaman College of Engineering (Autonomous), Hyderabad, Telangana India; 4 Department of CS&IT, Siksha ‘O’ Anusandhan (Deemed to be University), Bhubaneswar, Odisha India; 5 Department of Computer Science and Engineering, Sri Venkateswara College of Engineering and Technology (Autonomous), Chittoor, AP India; 6 School of Computer Science Engineering Systems, Vellore Institute of Technology, Vellore, India; 7 Department of Information Technology, Manipal Institute of Technology Bengaluru, Manipal Academy of Higher Education, Manipal, India

Retraction of: *Scientific Reports* 10.1038/s41598-025-04072-7, published online 01 July 2025

The Editors have retracted this Article.

After publication, concerns were raised regarding the presence of nonstandard vocabulary, the incorrect definitions of key evaluation metrics, insufficient description of dataset use, and performance values that could not be reconciled with the information provided. The Editors requested explanations, the code underlying the study, and additional methodological information on how the dataset was partitioned and what safeguards were put in place to prevent data leakage, but the responses from Authors did address the concerns raised. The Editors therefore no longer have confidence in the results and conclusions of this Article.

Rourab Paul agrees with the retraction. Bibhuprasad Sahu and J Shreyas disagree with this retraction. Amrutanshu Panigrahi, Abhilash Pati, Ajit Kumar Nayak, Subrata Chowdhury, and Ramya Govindaraj did not respond to the correspondence from the Editors about this retraction.

